# A Hybrid Deep Transfer Learning Framework for Delamination Identification in Composite Laminates

**DOI:** 10.3390/s25030826

**Published:** 2025-01-30

**Authors:** Muhammad Haris Yazdani, Muhammad Muzammil Azad, Salman Khalid, Heung Soo Kim

**Affiliations:** 1 Department of Mechanical Engineering, Dongguk University-Seoul, 30 Pildong-ro 1-gil, Jung-gu, Seoul 04620, Republic of Korea; mharisyazdani@dgu.ac.kr (M.H.Y.); muzammilazad@dgu.ac.kr (M.M.A.); 2 Department of Mechanical, Robotics and Energy Engineering, Dongguk University-Seoul, 30 Pildong-ro 1-gil, Jung-gu, Seoul 04620, Republic of Korea; salmankhalid@dgu.ac.kr

**Keywords:** vibration signals, delamination detection, delamination identification, transfer learning, deep learning, hybrid model

## Abstract

Structural health monitoring (SHM) has proven to be an effective technique to maintain the safety and reliability of laminated composites. Recently, both deep learning and machine learning methodologies have gained popularity in sensor-based SHM. However, machine learning approaches often require tedious manual feature extraction, while deep learning models require large training datasets, which may not be feasible. To overcome these limitations, this study presents a hybrid deep transfer learning (HTL) framework to identify delamination in composite laminates. The proposed framework enhances SHM performance by utilizing pre-trained EfficientNet and ResNet models to allow for deep feature extraction with limited data. EfficientNet contributes to this by efficiently scaling the model to capture multi-scale spatial features, while ResNet contributes by extracting hierarchical representations through its residual connections. Vibration signals from piezoelectric (PZT) sensors attached to the composite laminates, consisting of three health states, are used to validate the approach. Compared to the existing transfer learning approaches, the suggested method achieved better performance, hence improving both the accuracy and robustness of delamination detection in composite structures.

## 1. Introduction

Compared to metals, laminated composites offer advanced mechanical properties and significant weight reduction, making them highly advantageous for various applications [1]. These benefits have led to their widespread use across mobility and engineering industries [1,2,3]. However, owing to their orthotropic characteristics, laminated composites are prone to various damage mechanisms, including delamination, fiber fracture, and matrix cracking [4,5]. Among these, delamination, as the leading cause of catastrophic failure in composite structures, is the most critical form of damage [6,7]. Delamination is one of the most critical forms of damage in composite structures, as it significantly compromises their structural integrity and load-bearing capacity. This failure mode, characterized by the separation of layers within the laminate, poses a substantial threat to the reliability and safety of composite materials in high-performance applications [8]. Data-driven SHM systems have recently emerged as effective tools to maintain structural stability and streamline maintenance operations [9,10]. However, due to the internal nature of delamination, detecting damage in composites remains challenging, and the hidden nature of defects necessitates the application of SHM techniques to identify and evaluate damage that is not visible [11]. In consequence, various SHM methods and non-destructive evaluation (NDE) techniques have been explored to address this critical challenge in composite laminates [12].

Although these methods are effective, they often require significant operational skills to be successfully implemented, which are, in general, expensive, time-consuming, and complex to perform. In addition, the inspection process usually requires composite structures to be taken out of service and relies on customized signal-generating apparatus, resulting in further operational interruptions [1]. Considering this, vibration-based monitoring approaches have emerged as viable and promising alternatives for the SHM of composite materials. The direct integration of vibration sensors into composite structures, along with the measurement of their dynamic characteristics, makes vibration-based structural health monitoring technology an efficient and reliable solution [13,14,15]. However, they frequently face significant challenges in obtaining comprehensive vibrational data for composite structures under specific damaged conditions. Furthermore, the compromised structural integrity of the materials can introduce substantial risks during the data collection process.

To address the challenge of limited experimental data, numerical simulations and data augmentation techniques have been developed that generate sufficient data for different health classes [16,17]. These approaches expand training datasets by combining mathematical models for both healthy and damaged scenarios. While simulated data can provide adequate results to monitor composite laminates, the finite-element (FE) method requires significant expertise and intensive validation, which makes it time-consuming and challenging to generalize across diverse damage scenarios. In contrast, data augmentation enhances the reliability and applicability of SHM systems [18,19,20]. However, the temporal and spectral features inherent to vibrational data typically make the common data augmentation methods used in computer vision, such as cropping, flipping, and altering colors, unsuited to SHM applications [21,22,23].

This research aims to address the challenge of insufficient data for the SHM of composites without relying on simulation, data augmentation, or intensive experimentation. Transfer learning has become a widely adopted methodology in deep learning, particularly in scenarios with insufficient data. ImageNet pre-trained models have consistently demonstrated better performance, making them valuable for applications with limited datasets [24]. Transfer learning has revolutionized various fields by utilizing state-of-the-art models pre-trained on ImageNet, providing a strong foundation for various applications even in the case of data scarcity [25]. For example, Fotouhi et al. [26] utilized a pre-trained AlexNet model on visual inspection data to evaluate damage levels in laminated composites; when compared with the ResNet–50 TL model and other deep learning architectures, AlexNet outperformed other models but exhibited increased computational time. Similarly, Zhao et al. [27] proposed a VGG−16-based TL model for real-time damage identification, and Rai and Mitra [28] employed a pre-trained ResNet model with lamb waves for the SHM of laminated composites. An ResNet-based TL approach using digital image correlation was also developed for SHM applications [29]. Azad et al. [30] compared several TL models to detect fracture modes in composites, concluding that DenseNet outperformed GoogleNet in accuracy. However, despite its superior damage detection capabilities, YoloNet, due to its deep and complex architecture, demands significant computational resources [31]. In another study, Azad et al. [32] addressed the data scarcity issues using deep CNNs; their validation results showed improved performance of ResNet compared to a CNN model trained from scratch, Xception, and VGG-16 and VGG-19 models. Another study explored different lightweight transfer learning models for SHM in laminated composites and concluded that the EfficientNet model performed better compared to the NasNet and MobileNet models [33]. Consequently, while traditional transfer learning approaches are beneficial for SHM, they often rely on a single architecture, limiting their ability to capture complex damage characteristics in composite laminates.

This study therefore proposes a hybrid transfer learning (HTL) model that integrates the strengths of EfficientNet and ResNet to achieve enhanced feature extraction. The model uses the scalable efficiency of EfficientNet in parallel with the deep residual learning capabilities of ResNet. The contributions of this work are twofold: (a) addressing the data scarcity challenge in the SHM of composite structures and (b) improving delamination identification performance through a hybrid model. To effectively process raw vibration data from PZT sensors which are fixed to composite laminates, the continuous wavelet transform (CWT) is used to convert the data into scalogram images, capturing both spectral and temporal features. These scalograms are then used to train the HTL model; its performance is then comprehensively evaluated using metrics such as accuracy, precision, recall, F1-score, and the confusion matrix.

## 2. Proposed Methodology and Theoretical Background

### 2.1. The Proposed Methodology

**Figure 1:**
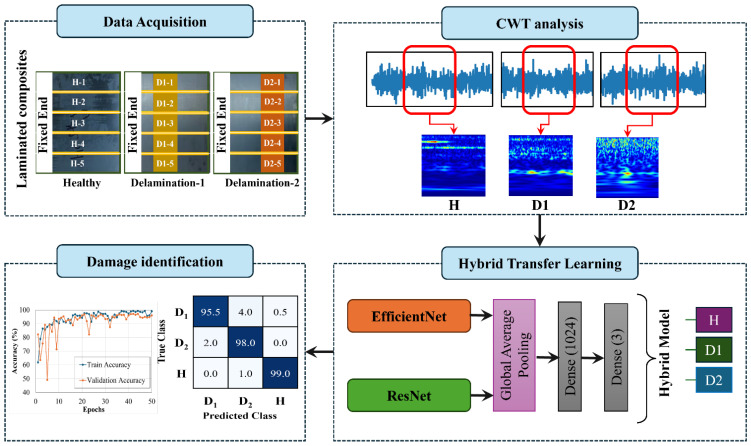
Overview of the hybrid SHM framework for composite laminates.

This research suggests the application of HTL models as an effective approach to the SHM of laminated composites. Figure 1 illustrates the suggested method (a) collecting data from experimental apparatus; (b) converting vibrational signals into scalogram images using CWT; (c) fine-tuning the HTL model on the scalogram images; and (d) assessing the health state of the laminated composites. Initially, vibration data are collected for three health states of the laminated composite samples. These health states consist of two cases of delamination damage (D1 and D2) and one healthy state (H). CWT analysis is used to produce images from the raw vibrational data. The third stage trains the HTL model using the image data, while, in the last stage, the trained HTL model predicts the health state of the composites using unseen test data. A confusion matrix and several additional matrices are derived from the confusion matrix to evaluate the proposed hybrid approach. Within the HTL model, the EfficientNet and ResNet layers are used to learn the characteristics of each class during training. The foundation for the proposed method is the framework of the HTL model, which consists of CNN layers sequenced in effective order. Fine-tuning in the EfficientNet architecture involves re-training selected layers of the pre-trained model on the delamination dataset, enabling the network to adapt its learned features to the specific task of delamination identification. The following section describes the employment of the transfer learning models of EfficientNet and ResNet to develop the HTL model.

### 2.2. Theoretical Background of the Applied Algorithms

#### 2.2.1. ResNet50V2 Model

ResNet50 and ResNet100 are deep learning models that are trained on the ImageNet dataset, which contains over a million labeled images across 1000 categories, while RNV2 is an upgraded version of these models [34]. The creation of the ResNet model was influenced by the pyramidal cells of the cerebral cortex, as this model employs shortcuts that bypass specific connections or layers. ResNet typically functions using shortcut connections to skip two- or three layers, where the skipped layers are composed of batch normalization and non-linear activation functions. By allowing the network to focus on residual mappings, this design helps streamline learning, simplifying the training process. However, using highway nets, it is possible to investigate the weights of skipped levels by employing an additional weight matrix. Allowing gradients to pass across the network can reduce the vanishing gradient problem, which, in turn, increases the training stability and accelerates the convergence [35]. The basic equation for a single residual block in ResNet50v2 can be expressed as Equation (1):

(1) $$y=F\left(x,\{W_{i}\}\right)+x$$

where x is the input, and y is the output to the residual block, while $F\left(x,\{W_{i}\}\right)$ is the residual mapping learned by the block, represented by a stack of layers with weights $\left\{W_{i}\right\}$.

**Figure 2:**
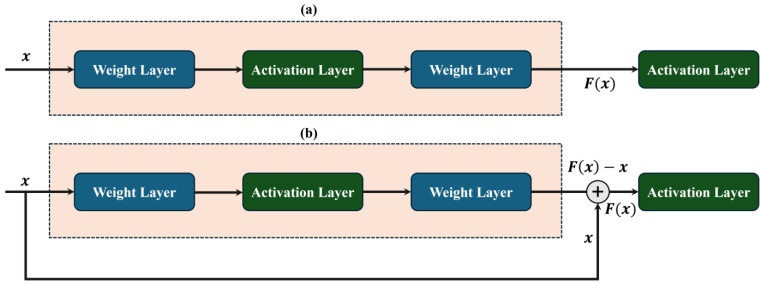
Schematic comparison of (**a**) normal and (**b**) residual blocks.

**Figure 3:**
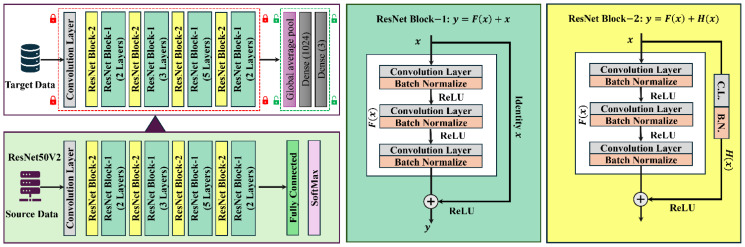
A detailed schematic of the ResNet50V2 model and the process of transferring information to the target domain.

The residual block (RB) is the core component of the ResNetV2 model; hence, Figure 2 compares the RB and normal block. Presume input data x with the underlying features $F\left(x\right)$, which are an input to the activation function. In Figure 2, the dotted box represents the block that learns the features. In the standard block approach, the model would learn the features as F(x). However, with a residual block, the model instead learns the features F(x)−x. This simplifies the learning task, as the feature can be represented by the identity function F(x)=x, which implies that the weights and biases of the upper layer are set to zero, making the learning process easier. Based on these properties, the set of layers is known as the RB. The solid line in Figure 2 denotes the flow of features; but, in the RB, a shortcut or skip connection links the input data x directly to an addition operator, effectively bypassing a series of layers. The shortcut connection helps speed up the propagation process across layers that are supported by the residual connection. The propagation links between blocks have been modified in RNV2, the expanded version of ResNet50. Thus, the core concept of RNV2 is to find connections that bypass many layers. By utilizing transfer learning phenomena with a pre-trained network, the suggested RNV2 model can overcome the limited data issue. Figure 3 shows the general layout of the RNV2 network. ResNet Block–1 (RNB1) and ResNet Block–2 (RNB2) are the two different types of block structures that the design includes. Each block consists of a CNN-based architecture that includes convolutional layers for feature extraction, batch normalization to stabilize and accelerate training, ReLU activation for non-linear transformations, and shortcut residual connections to enable efficient gradient flow and mitigate vanishing gradient issues. RNB1 and RNB2 comprise an identity function F(x)=x, along with convolution and batch normalization layers within the residual connection, respectively.

The architecture utilizes multiple stacked RNB1 and RNB2 blocks to enable autonomous feature extraction from the input data. Following this, the weights and learned features of the pre-trained model developed on a large source dataset are transferred to the target domain. To adapt the model to the specific task, the limited target dataset is used to fine-tune the remaining blocks. This fine-tuning process enhances the model performance by optimizing its parameters for the unique characteristics of the target domain. The additional fine-tuning layers in each model contain a global average pooling (GAP) layer, a dense layer, and a classification layer with Softmax activation. Figure 3 shows that, after being pre-trained on a source dataset, the RNV2 model demonstrates knowledge transfer through its application to the target dataset. The diagram highlights the transfer process: layers with fixed weights are marked with locked icons, indicating that these network blocks are frozen and remain unchanged during training on the target dataset. In contrast, the unlocked icons represent blocks that are fine-tuned, allowing their weights to be adjusted for the specific task. This selective fine-tuning approach ensures that the model leverages the robust features learned during pre-training while effectively adapting to the nuances of the target domain, leading to improved performance.

#### 2.2.2. EfficientNet Model

EfficientNet is a prominent deep learning model that is renowned for its exceptional performance in image classification problems. Instead of using the ReLU, which is the activation function that conventional CNNs use, EfficientNet uses the Swish activation function, which results in improving the performance of the network [36]. By minimizing the number of parameters while maintaining a high performance, EfficientNet represents a significant advancement in the design of computationally efficient models. Its efficiency is achieved from the compound-scaling approach, which uniformly scales the depth, width, and resolution of the model. This method optimizes performance without being computationally intensive, maintaining a balance between resources and model accuracy. This scaling process involves formulating scaling factors across multiple dimensions (depth, width, and resolution) while adhering to predefined resource limitations [33,34]. By systematically balancing these dimensions, EfficientNet achieves superior efficiency and scalability compared to traditional scaling methods that modify only a single parameter. Initially, the mobile inverted-bottleneck convolution (MBConv) was introduced in the MobileNetV2 model, which is a core element of EfficientNet [37]. Compared to expansion layers, MBConv layers greatly reduce the number of floating-point operations per second (FLOPS) by first expanding and then compressing channels to connect bottlenecks with fewer channels. The depth-wise separable convolutions used in this architecture reduce the required calculations by a factor of $k^{2}$, where k denotes the kernel size [38]. In the compound-scaling approach, the dimensions of the model are all scaled uniformly by adopting a coefficient ψ. Under fixed resource restrictions, grid search is used to determine the constants α, β, and γ that govern this scaling [38]. The scaling equations are as follows:

(2) $$depth::\;d=\alpha^{\psi }\;width::\;w=\beta^{\psi }\;resolution::\;r=\gamma^{\psi }\;\alpha ,\;\beta ,\gamma \geq 1$$

**Figure 4:**
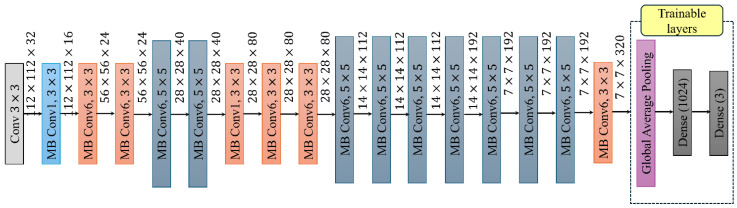
Architecture of the EfficientNet-based transfer learning model.

While ψ regulates overall scaling, these constants (α, β, γ) define how extra resources are distributed across network dimensions. The grid search is initially performed with ψ=1, assuming the doubling of available resources, to determine the optimal values for α, β, and γ. Secondly, these values are fixed, and the larger models are obtained by scaling the baseline network using various ψ values. This unique scaling technique allows EfficientNet to efficiently attain greater performance [38]. Figure 4 displays the architectural characteristics of the EfficientNet–B0 baseline model that is used in this study. The text at the bottom of the figure illustrates the order in which the MBConv layers are applied, while the text at the top of the figure illustrates the dimensions of the output feature map that is generated after each layer. The ImageNet dataset is used to pre-train the EfficientNet model; subsequently, three additional layers are incorporated into the pre-trained model to fine-tune it for the target data of laminated composites. The additional fine-tuning layers in each model contain a GAP layer, a dense layer, and a classification layer with Softmax activation. The GAP layer is used to decrease the features that the EfficientNet layers have extracted, by averaging each feature map into a single value. All three newly added layers in the EfficientNet model are trainable to allow them to adapt to the target vibration-based scalograms of composite laminates, while the weights of the preceding layers remain fixed to preserve the knowledge learned from the pre-trained model.

#### 2.2.3. The Hybrid Efficient–ResNet Model

The hybrid model combines the strengths of the ResNet-based RNV2 model and the EfficientNet model to create a powerful framework to address the challenges of SHM in laminated composites. Integration of these two architectures allows the hybrid model to use their individual capabilities while overcoming their respective limitations, resulting in a robust and efficient approach to feature extraction and classification. The hybrid model utilizes the complementary strengths of ResNet’s deep hierarchical feature extraction and EfficientNet’s parameter-efficient design. However, the integration is performed for one deeper model (ResNet) and one lightweight model (EfficientNet). Thus, the overall computational requirements are not massively increased due to the use of one lightweight model rather than hybridizing two deeper models. The integration process involves utilizing the pre-trained weights of both models while fine-tuning them on the target dataset to ensure optimal performance. The additional fine-tuning layers in each model contain a GAP layer, a dense layer, and a classification layer with Softmax activation. Together, these components enable multi-scale damage patterns and complex structural behaviors characteristic of laminated composite structures to be addressed by the hybrid model.

## 3. Validation of the Proposed Methodology

### 3.1. Data Acquisition

**Figure 5:**
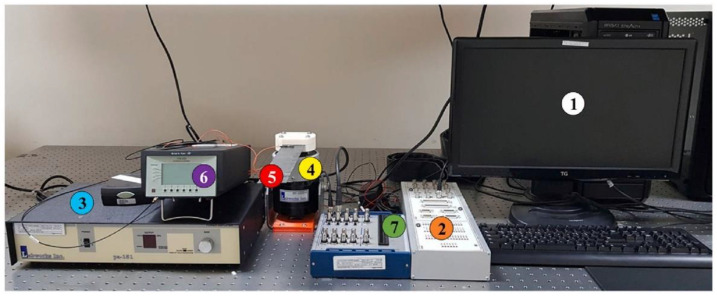
Experimental setup for data acquisition: (1) LabView PC, (2) excitation DAQ, (3) amplifier for shaker, (4) shaker, (5) composite sample, (6) amplifier for accelerometer, and (7) data acquisition system [32].

In this study, carbon fiber prepreg [0/90/0/90]_s_ was used to manufacture composite samples. The samples were produced by a hot-press compression modeling technique. The developed composites consisted of three health conditions: the healthy state (H), the delamination–1 (D1) state, and the delamination–2 (D2) state. Delamination was introduced in the middle plane of the composites by incorporating a Teflon film, which acted as a separation layer to simulate interlayer damage. In the experiment, both delaminations were of identical dimensions, with D1 situated nearer the clamped end, while D2 was positioned near the free end. This was performed in the cantilever beam configuration, and to address manufacturing and experimental uncertainties, five samples were tested for each condition. Figure 5 shows the experimental setup, which consisted of a data collection (DC) system and an excitation and vibration system. The excitation system used MATLAB Simulink and included a LabVIEW PC that produced random signals. These random signals were sent to the shaker with the help of an amplifier and the data that were used as input excitation. The vibration apparatus was used to apply shaker excitation to composite specimens mounted in a cantilever beam setup. The main component of the DC system was the accelerometer, which was connected to the free end of the composite to produce random signals from the three different health states. In addition, an amplifier was used to amplify the obtained signal for recording.

**Figure 6:**
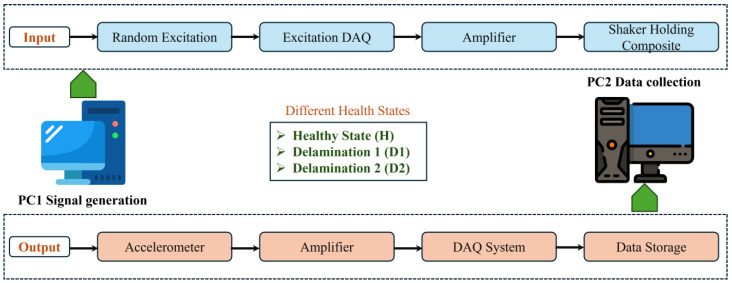
Diagram showing the experimental setup used to collect vibrational data from laminated composites.

Vibration data were collected over 15 s from five samples, each representing a distinct structural condition, with a sampling rate of 2.5 kHz to ensure accurate temporal resolution of the signal. To improve the dataset diversity, 10 random responses were gathered from each sample. Those ten random samples were combined with the five samples to represent each health condition, which resulted in the generation of fifty scenarios for each condition, thus exhibiting significant diversity. The gathered vibration data were originally in a one-dimensional format, which could be transformed into two-dimensional images. The schematic diagram of data acquisition in this study is shown in Figure 6.

### 3.2. CWT Analysis

**Figure 7:**
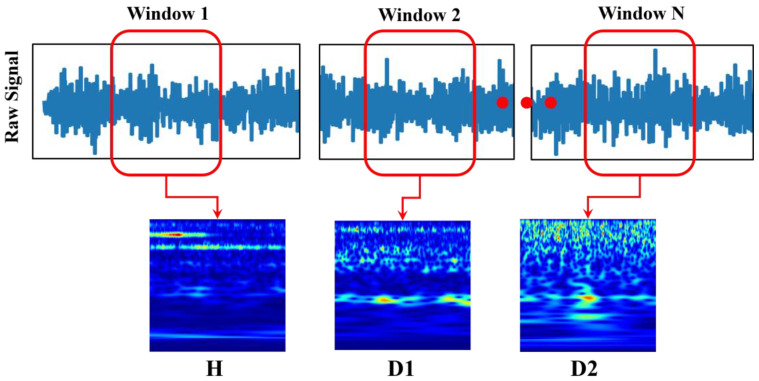
CWT processing of vibrational data into scalograms.

The CWT of a signal provides a time–frequency representation, often visualized as scalograms. This transform helps analyze both the temporal and spectral components of the signal over time [39]. Unlike a spectrogram, a scalogram is particularly helpful in analyzing signals in engineering applications where characteristics vary across scales. It is well suited to identify patterns that involve gradual changes interrupted by sudden, brief occurrences. The CWT method enhances temporal localization for events that are shorter and occur with a higher frequency, while it provides support for frequency tracking for events that are longer and occur with a lower frequency. To generate the CWT, the signal was resampled using a wavelet function that was systematically time-shifted and scaled, allowing for the extraction of localized time–frequency features from the data. To achieve scalability and transitional operations, a wavelet was utilized during the CWT process. CWT scaling dynamically adjusted the wavelet function by stretching and shrinking it. By stretching the wavelet, it becomes longer and focuses on capturing extended, low-frequency signals. Conversely, compressing the wavelet creates a shorter, higher-frequency waveform that is suited to identifying fast-changing high-frequency events [40]. In this research, using a window size of 1875 sequential sample points from the vibration data, CWT converted the 1D vibration data into 2D scalogram images [32]. Through this process, 1000 scalogram images were obtained for each class, resulting in a total of 3000 images, as Figure 7 shows.

### 3.3. HTL Model Development and Performance Evaluation

**Figure 8:**
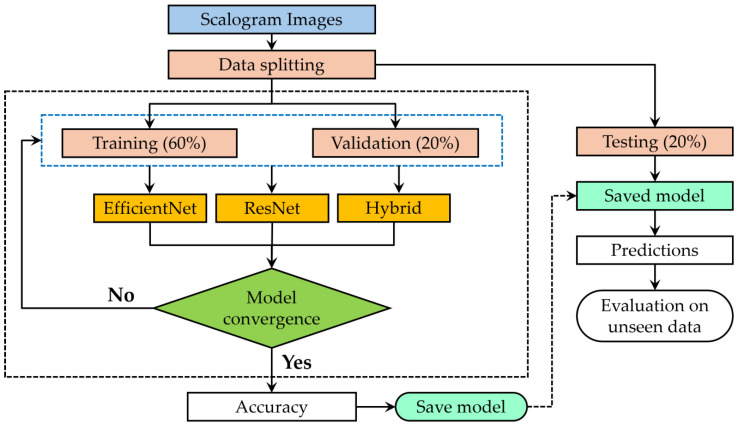
The flowchart illustrating the working of all three models.

Figure 8 shows a flowchart that describes the workflow for training, validating, and testing all the discussed machine learning models using scalogram images as the input. The method was initiated by partitioning the dataset into three segments: 60% assigned for training, 20% for validation, and 20% for testing. Thus, the number of images in the training, validation, and testing groups was 600, 200, and 200, respectively. The models were trained using three architectures during the training phase: EfficientNet, ResNet, and a hybrid model. The input scalogram images were resized to 224×224 pixels to match the model requirements of the pre-trained model. All model outputs consisted of a dense layer with three neurons, each representing one of the three target classes. Validation data were used to monitor and evaluate the model performance during the training phase. The flow included a decision point, where the convergence of the models was checked. If the models failed to converge, the training and validation cycle was repeated. Once convergence was achieved, the model accuracy was measured, and the best-performing model was saved. The saved model was subsequently assessed with the testing dataset to generate predictions, which were analyzed to evaluate the model efficacy on an unseen dataset. This structured approach guaranteed the creation of a reliable and precise model.

### 3.4. Performance Assessment Metrics

Accuracy, precision, recall, F1-score, and the confusion matrix are the metrics that were used to determine the effectiveness of the transfer learning models. The following mathematical formulae represent these metrics using true-positive ($T_{p}$), true-negative ($T_{n}$), false-positive ($F_{p}$), and false-negative ($F_{n}$) predictions:

(3) $$Accuracy=\frac{T_{p}+T_{n}}{T_{p}+T_{n}+F_{n}+F_{p}}$$

(4) $$Precision=\frac{T_{p}}{T_{p}+F_{p}}$$

(5) $$Recall=\frac{T_{p}}{T_{p}+F_{n}}$$

(6) $$F1\text{-}score=\frac{2T_{p}}{2T_{p}+F_{n}+F_{p}}$$

The confusion matrix was also used to estimate the SHM performance of the transfer learning models, presenting an overview of the actual and expected outcomes.

## 4. Results and Validation

According to the information presented in Section 2 of this research, transfer learning models were applied for the SHM of the laminated composite. First, the EfficientNet and ResNet models were each developed individually. Second, the HTL model was developed by combining the features from both models into a single model. The weights of all layers in the pre-trained model were kept unchanged, and a global average pooling (GAP) operation was employed to reduce feature map dimensions. A dense layer was then added before the final classification layer to facilitate task-specific predictions. The target scalogram data from laminated composites were used to re-train (fine-tune) the newly added top three layers of the model, allowing these layers to adapt to the specific characteristics of the new dataset, while the pre-trained layers remained unchanged. Thus, only the layers on top were trained using random weight initialization and feature mappings from the EfficientNet, ResNet, and HTL architectures. A key advantage of the proposed strategy is that model training is only performed once, which reduces the requirements for repeated training sessions, saving time and computational resources. Additionally, the efficacy of the suggested HTL model was assessed relative to the individual transfer learning models of ResNet and EfficientNet. With the same number of layers applied on top, all transfer learning models were trained for 50 epochs for fair comparison.

**Figure 9:**
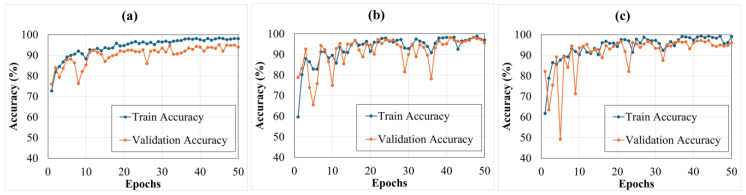
Training curves of transfer learning models (**a**) EfficientNet, (**b**) ResNet, and (**c**) HTL, showing performance over the number of epochs.

Figure 9 shows the validation curves that were obtained during the training process of transfer learning models that show the training. The curves indicate that all models converged within 50 epochs, highlighting their capacity for rapid learning. The training process was considered converged when the validation accuracy demonstrated minimal improvement over successive epochs. To ensure optimal performance while preventing overfitting, an early-stopping criterion was applied. Specifically, training was stopped after 10 consecutive epochs without an improvement in validation accuracy. This approach strikes a balance between achieving high model accuracy and maintaining computational efficiency, ensuring a robust and efficient training process. The EfficientNet model showed training and validation accuracy of (99.78 and 94.83)%, respectively; however, it exhibited a considerable degree of overfitting, evident in the significant disparity between its training and validation accuracies. In comparison, the ResNet model showed training and validation accuracy of (98.61 of 95.50)%, respectively, revealing a reduced level of overfitting; this indicates a more balanced performance between the training and validation phases. The proposed HTL model outperformed both, with a training and validation accuracy of (99.89 and 96.00)%, respectively; this model demonstrated improved convergence and the least overfitting among the three, making it a robust option. Overall, while EfficientNet demonstrated superior performance for the SHM of composite laminates in scenarios with limited training resources, the ResNet and HTL models showed better generalization and reduced overfitting. The hybrid approach is particularly suited to laminated composites, which often exhibit multi-scale damage features. EfficientNet excels at identifying smaller-scale details, while ResNet captures broader structural patterns, enabling the hybrid model to address the diverse nature of composite material behavior.

**Figure 10:**
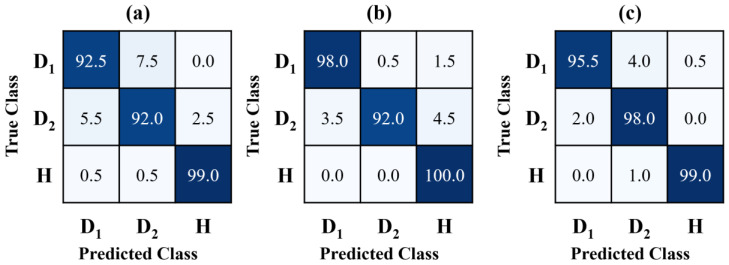
Illustration of the confusion matrix for (**a**) EfficientNet, (**b**) ResNet, and (**c**) HTL models, using the unseen test datasets.

To evaluate their generalization and performance, the HTL models were assessed on unseen data, using the performance metrics detailed in Section 3.4 for better estimation. Evaluating unseen data is crucial to assess the generalization capability of deep learning models and can be considered the model’s ability to perform effectively on new, unseen data beyond the training set. Overfitting occurs when a model achieves high performance on the training data but fails to generalize to unseen test data, leading to poor performance in real-world scenarios. By evaluating the model on unseen data, researchers can identify overfitting and ensure the model’s performance in real-world scenarios. The EfficientNet, ResNet, and HTL models showed a testing accuracy of (94.50, 96.67, and 97.50)%, respectively. The classification performance of all three transfer learning models was evaluated using confusion matrices (CMs), as shown in Figure 10. A confusion matrix provides a tabular visualization of model predictions, with diagonal elements representing correct classifications (true-positives and true-negatives) and off-diagonal elements indicating misclassifications (false-positives and false-negatives). For the EfficientNet model, the diagonal cell values were notably reduced, indicating a lower accuracy in classifying all health states, particularly delamination cases D1 and D2. The off-diagonal cell values for the damaged states D1 and D2 were higher than their corresponding diagonal values, reflecting frequent misclassifications. This highlights that the EfficientNet model struggled to accurately identify delamination scenarios compared to the healthy state. In contrast, the ResNet model demonstrated improved classification accuracy across all health states. Notably, the classification accuracy for delamination D1 was significantly higher, achieving a performance of 98%. The proposed HTL model further outperformed both EfficientNet and ResNet, achieving superior classification accuracies for all health states: 99.0% for the healthy state, 95.5% for delamination D1, and 98.0% for delamination D2. Moreover, the HTL model exhibited greater reliability, as evidenced by the smaller variation in classification accuracy across different health states. Although some degree of uncertainty remained in differentiating between delamination types D1 and D2, likely due to uniform damage patterns in the composite laminates, the HTL model demonstrated fewer instances of misclassification compared to EfficientNet and ResNet. Additionally, Table 1 presents other evaluation metrics, including precision, recall, and F1-score, for each health state. The HTL model achieved high precision values for D1, D2, and H, recorded at (97.95, 95.15, and 99.50)%, respectively. Precision reflects the ratio of true-positives to total positive predictions, indicating the model’s ability to avoid false-positives. The HTL model also recorded superior recall values of (95.50, 98.00, and 99.00)% for D1, D2, and H, respectively, which measure the proportion of true-positives correctly identified by the model. These results highlight the HTL model’s ability to achieve a balanced and superior performance across all health states, making it more reliable and effective than the existing transfer learning models in identifying delamination scenarios.

The F1-score, defined as the harmonic mean of precision and recall, integrates both precision and recall into a single value, offering a comprehensive measure of model performance. In the case of D1 and D2, the HTL model achieved maximum F1-scores of (96.71 and 96.55)%, respectively, while the F1-score for H was 99.25%. These findings indicate that the pre-trained hybrid Efficient–ResNet model showed better performance than the other transfer learning models for the SHM of the laminated composite structures.

In Table 2, the performance of the proposed HTL model is compared with the CNN model trained from scratch and pre-trained models such as Xception, VGG-16, VGG-19, NASNetMobile, MobileNet, and ResNet. It is evident that the pre-trained models showed improved performance. Based on the results, it is better to use pre-trained models such as ResNet and EfficientNet and their hybrid model for SHM in laminated composites.

This research reveals the potential of HTL models for the SHM of laminated composites. The suggested HTL approach makes it possible to effectively evaluate laminated composites that exhibit comparable dynamic characteristics and have the same delamination at different locations. Future research should study the application of these techniques to unsupervised learning. This approach has the potential to broaden the methodology for the SHM of laminated composites, particularly in scenarios where labels are not known beforehand.

## 5. Conclusions

This research presented a hybrid model based on transfer learning for the SHM of composite laminates. The suggested approach was validated by the vibration signals gathered from experimentation. The raw vibration signals were transformed into time–frequency scalogram images by CWT analysis. Firstly, the traditional transfer learning models of ResNet and EfficientNet were assessed, and depending on the previously discussed models, a hybrid model termed the HTL model was proposed. The findings showed that ResNet and HTL models performed better during training, while the EfficientNet model was prone to slight overfitting. This resulted in F1-scores for the D1, D2, and H states, which were (96.71, 96.55, and 99.25)%, respectively, after fine-tuning on the target dataset. Therefore, using the HTL model’s architecture, the proposed method showed improved generalization capabilities. As a result, the issue of training data limitations was effectively addressed, especially in cases where laminated composites could not provide sufficient data for delaminated scenarios. Therefore, the proposed method can be further fine-tuned to accommodate various other composite structures, because fine-tuning can result in the robustness of the SHM of laminated composites. CNNs, while effective for spatial feature extraction, lack inherent temporal modeling capabilities. In this study, the temporal information encoded along the time axis of scalograms was utilized as spatial features, enabling the CNN-based hybrid model to process spatiotemporal patterns effectively. The current work focuses on delaminations of a uniform size at various locations within composite laminates. Future studies could explore delaminations of varying sizes and positions and incorporate advanced temporal modeling techniques, such as dilated causal convolution, to further enhance temporal awareness. While this study is limited to delamination-related damage, the proposed framework can be adapted to detect other defects, such as matrix cracking or fiber breakage, with appropriate modifications and targeted datasets. This would broaden its applicability and enhance the reliability of composite structure assessments.

## Figures and Tables

**Table 1:** Comparison of the different HTL models using different performance metrics.

Health State	HTL Model	Precision (%)	Recall (%)	F1-Score (%)
D1	EfficientNet	93.91	92.50	93.20
	ResNet	96.55	98.00	97.27
	Proposed HTL	97.95	95.50	96.71
D2	EfficientNet	92.00	92.00	92.00
	ResNet	99.46	92.00	95.58
	Proposed HTL	95.15	98.00	96.55
H	EfficientNet	97.54	99.00	98.26
	ResNet	94.34	100.00	97.09
	Proposed HTL	99.50	99.00	99.25
Average Performance	EfficientNet	94.48	94.50	94.49
	ResNet	96.78	96.67	96.65
	Proposed HTL	97.53	97.50	97.50

**Table 2:** Comparison of the CNN model trained from scratch with pre-trained models and the proposed HTL model.

HTL Model	Accuracy (%)	Precision (%)	Recall (%)	F1-Score (%)
CNN model [32]	40.33	40.66	40.33	40.33
Xception [32]	84.67	85.00	84.33	84.67
VGG-16 [32]	93.67	93.67	93.67	93.67
VGG-19 [33]	91.33	91.33	91.33	91.33
NASNetMobile [33]	83.67	82.44	84.50	83.46
MobileNet [33]	92.50	90.38	94.00	92.16
ResNet	96.67	96.78	96.67	96.65
EfficientNet	94.50	94.48	94.50	94.49
Proposed HTL	97.50	97.53	97.50	97.50

## Data Availability

The original contributions presented in this study are included in the article. Further inquiries can be directed to the corresponding author.
